# The shape language in application to the diagnosis of cervical vertebrae pathology

**DOI:** 10.1371/journal.pone.0204546

**Published:** 2018-10-11

**Authors:** Marzena Bielecka, Rafał Obuchowicz, Mariusz Korkosz

**Affiliations:** 1 Chair of Geoinformatics and Applied Computer Science, Faculty of Geology, Geophysics and Environmental Protection AGH University of Science and Technology, Cracow, Poland; 2 Department of Radiology, Jagiellonian University Medical College, Cracow, Poland; 3 Division of Rheumatology, Departement of Internal Medicine and Gerontology, Jagiellonian University Hospital, Cracow, Poland; Massachusetts Institute of Technology, UNITED STATES

## Abstract

In this paper the possibility of classification of X-ray images of the cervical vertebrae is studied. The images should be classified into one of the following classes—the images of healthy vertebrae and the images of vertebrae with syndesmophytes. The vertebra contours, described unambiguously by using the generalized shape language, are the basis of the analysis. As a result, the contour is represented as a chain of sinquads that determine switches. The found switches are the characteristic points of the analyzed contour. In these points additional features of the contour are determined. On the basis of these features two aforementioned classes of images are defined as fuzzy sets. Such an approach allows us to create a hierarchical algorithm of classification based on the syntactic and fuzzy description of the contour.

## Introduction

In recent years the number of medical examinations which consist in analyzing X-ray images of bones has increased rapidly [1]. In general, the images are analyzed in two aspects: the analysis of bone density and the analysis of bone structures. It should be also mentioned that the width of joints is analyzed as well, and this topic has been worked out relatively well [2–6]. Both the aforementioned aspects of bone images analysis provide crucial pieces of information about pathological changes and, as a consequence, play an important role in diagnosis and assessment of the disease progress—see the next section for more details. According to the aforementioned large number of X-ray medical images, the methods of their automatic analysis are being sought intensively. In particular, the following topics are studied:

- context-based retrieval of medical images [1, 7, 8],
- automatic localization of cervical vertebrae [9, 10],
- analysis of contours of finger bones [11–18],
- application of image languages to analysis of radiological palm images [19, 20],
- shape representation based on statistical methods [21].

The syntactic analysis of bone contours is among the methods of studies which are based on geometrical features of the examined object. The syntactic pattern analysis, called sometimes syntactic pattern recognition, and the syntactic scene analysis are performed on the basis of a formal, structural representation of the studied object or scene [22, 23]. In the syntactic object analysis, the simplest elements from which the object is constructed—so-called primitives—and the structural relations between primitives are studied, whereas in the scene analysis the spatial relations between objects are analyzed, as well. In the analysis of medical X-ray images, bone structures are analyzed both on the level of a single pattern analysis and on the level of the scene analysis. In the first case, the shape, usually the contour of a bone, is examined, whereas in the second case the spatial relations among the bones are investigated. For both cases the syntactic methods are used—string languages are used for the contour analysis [12–14, 24] whereas graph languages are applied to the analysis of the anatomical structures constituted by a group of bones, for instance in a hand [19, 20]. Since the biological structures are irregular, the syntactic approach is frequently aided by fuzzy methods. The perspectives of assessing the disease progress are discussed as well. The proposed syntactic approach, based on the shape language [25, 26], is combined with fuzzy methods. As a result, a hierarchical analysis is proposed.

Syntactic methods have their own specific nature. The fact that these kind of methods are extremely sensitive to the pattern distortions is among the crucial ones. Therefore, the analyzed patterns, the bone contours in the considered case, should be obtained from the carefully preprocessed images. The effective preprocessing of medical X-ray images is difficult and the achieved results are far from the satisfactory ones [15]. On the one hand muscles, bones, cartilage, and tendons have various coefficients of X-ray absorption. On the other hand, they cover mutually in a complex way. Therefore, the preprocessing works well with soft-tissue objects but it is poor with bones [27]. This results in creating false bone edges and the discontinuities of contours [28]. That, in turn, causes that such changes as erosions, osteophytes and syndesmophytes are difficult to be detected in early stages of development. Furthermore, it is reported that the contrast of spine X-ray digitized images is low and, as a result, the image quality is poor [1, 7, 8]. The aforementioned problems make the preprocessing of the X-ray image a challenging task. The images used in the studies described in this paper were preprocessed by using Statistical Dominance Algorithm (SDA, for abbreviation) that is dedicated to preprocessing medical images [29, 30]. Application of the algorithm resulted in obtaining contours that have sufficient quality to be the basis for application of the proposed approach.

## Clinical background

**Fig 1:**
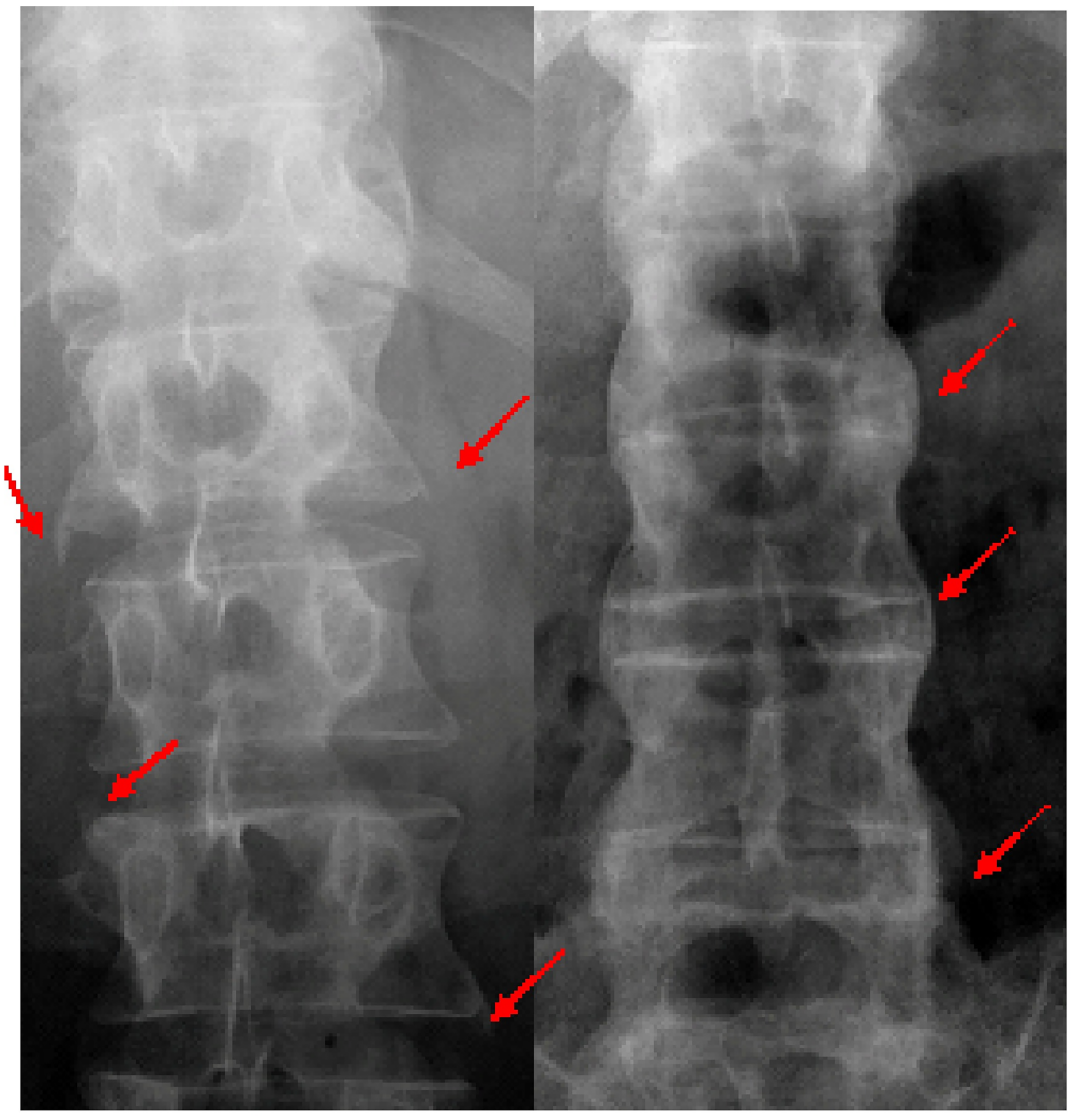
Syndesmophytes in the spine. On the left: Growing syndesmophytes in the spine of a 30-year old male with ankylosing spondylitis. On the right: Multiple syndesmophytes, bridging adjacent vertebral bodies in the spine of a 37-year male with ankylosing spondylitis, responsible for the so called bamboo spine image on X-ray.

Let us present a clinical motivation for the presented studies [31, 32]. Spondyloarthritis (SpA, for abbreviation) represents the second most prevalent inflammatory rheumatic group (ca. 2% in Caucasians) and it is characterized by chronic inflammation and structural damage involving the axial and peripheral skeleton. SpA in adults consists of several diseases, i.e. ankylosing spondylitis, psoriatic arthritis, reactive arthritis, arthritis in inflammatory bowel diseases and undifferentiated spondyloarthritis. All the diseases share similar axial (sacroiliitis, spondylitis) or peripheral (arthritis, enthesitis, dactylitis) manifestations. The disease is a significant burden both for the health system and for an individuals quality of life because the patients have several unfavorable consequences of chronic inflammation and structural damage to the skeleton. Formation of syndesmophytes in the vertebral bodies of the spine—see Fig 1—is the key issue in structural damage in SpA. Syndesmophytosis is also referred to as osteogenesis or osteoproliferation and from a pathophysiologic point of view, it is a new bone formation. Therefore, in SpA the interaction between chronic inflammation and bone tissue results in a new bone formation that is responsible for the remodeling of the spine, which becomes stiff and inflexible. The remodeling of the spine is associated with decreased quality of life, including daily activities, employment, family life and leisure time. The imaging assessment of growing and established syndesmophytes is of supreme importance for planning lifelong therapy. Fast progressing syndesmophytes requires complex and aggressive therapy, including non-steroidal anti-inflammatory drugs and biologic agents, eg. Tumour Necrosis Factor inhibitors which attenuate the chronic inflammation may inhibit or reduce the osteogenesis. Different scoring methods based on X-ray imaging have been developed for assessing structural damage in SpA, mainly the formation of syndesmophytes. Currently, modified Stoke Ankylosing Spondylitis Spine Score (mSASSS, for abbreviation) is the only system that proved to be reliable and sensitive to change, and, therefore, it is preferred in clinical practice both for detecting and following the disease progression. Nevertheless, the minimal time interval to reveal the least significant change in the structural damage progression has been established for two years, which seems to be, an excessively long period for treatment decision making, including monitoring of the effectiveness of the treatment. In other words, continuation, modification or discontinuation of expensive therapies of significant numbers of SpA patients should be based on the response-to-treatment assessment, by answering the question whether the therapy inhibits new bone progression. That is why new methods of osteogenesis follow-up are of significant importance, particularly if they could make the two-year-time interval of mSASSS assessment shorter.

## The state of the art

The methods of X-ray images computer investigations, based on the analysis of the shape of anatomical structures, are not used widely because of their complexity and sensitivity to distortions. In this section some examples of the analytical approaches of bone contours and the related problems in digital X-ray images are briefly recalled.

In the papers [9, 33] and [10] the problem of automatic localization of cervical vertebrae was considered. The presented approach is based on the generalized Hough transform, introduced in the paper [34] in order to detect curves which cannot be described by using an analytic formula, as in (simple) Hough transform. As a result, an arbitrary object in the image can only be recognized if it is encoded by its model which also represents the variability of the shape of the recognized object. This approach is invariant to scale and rotation as well as noise.

An important research stream concerns the description of the shape in the context of retrieval of medical images from database. The papers [7, 8] are examples of such studies. In the presented approach the vertebrae contours are encoded by using polygon approximation. In this method, the description of the shape of the analyzed contour is based on three mechanisms. Simplifying the shape description by keeping only relevant features is the first one. It is achieved by selecting the points which have the largest contribution to the shape. Representing the contour in the tangent space by using, so-called, turn function is the second one. The similarity of the contours, described by their turn functions, can be obtained by using similarity measurement which is the third mechanism the method is based on. These investigations were a significant contribution to shape-based retrieval techniques for biomedical images. There are a few other methods concerning shape description in order to retrieve content-based databases. Let us mention them very briefly. Some of them use shape properties such as elongation, perimeter, convexity and orientation, whereas others are based on invariant moments, and others are based on multi-scale shape representation—see [8, 35] and references given there. Another approach to shape description, proposed in [21], is based on statistical methods. Creation of a database of qualitative anatomical features, derived from the images and based on image characteristics, is the aim of the studies. The model shape is created as the mean value of the sample shapes. A grey-scale profile is the second component the method is based on. In the paper, the method is used to describe the shapes of vertebrae. The authors connect the problem of automatic localization of vertebrae in the X-ray image with the problem of automatic retrieval of medical databases. It should be stressed that the problems discussed above, i.e. shape description in order to create and retrieve content-based databases of X-ray images and automatic localization of anatomic structures in the X-ray images, though related to the topic considered in this paper, are, however, different from it. In this paper, we focus on analyzing a single vertebra contour in order to not only detect the pathological changes in bones such as osteophytes and syndesmophytes but also to create a tool which allows us to assess the progress of the disease. The authors intend to create a software tool that compares the pathological changes in a given vertebra by using a series of X-ray images taken, let us say, every half year for a given patient. This will allow the physician to assess the speed of the changes that take place in the spine. Such an assessment is crucial to judge whether the applied therapy is effective. The most related problem is considered in [1]. The method is based on the aforementioned polygon approximation approach. In the context of detection of the bone pathological changes, only these fragments of vertebra contour where the pathologies can occur are described. The contour has to be described in a maximally compressed way because the pathological changes detection is based on the retrieval of databases in which bone contours with the considered pathologies are stored. The method described in this paper—see Section Cervical vertebrae contours analysis—is based on syntactic analysis of the contour. The analysis is combined with fuzzy inference. Such an approach allows us to describe and analyze very precisely all types of the pathologies which manifest in the vertebra contour.

## The generalized shape language

**Fig 2:**
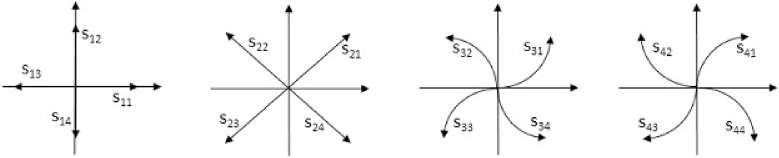
The shape language primitives.

The shape language, introduced by Jakubowski [36] as a syntactic tool for analysis of contours, was used by us as a theoretical starting point. In the shape language, sixteen primitives are defined—eight line segments and eight circle quadrants denoted as *s*_*ij*_, *i*, *j* ∈ {1, 2, 3, 4}, see Fig 2.

**Fig 3:**
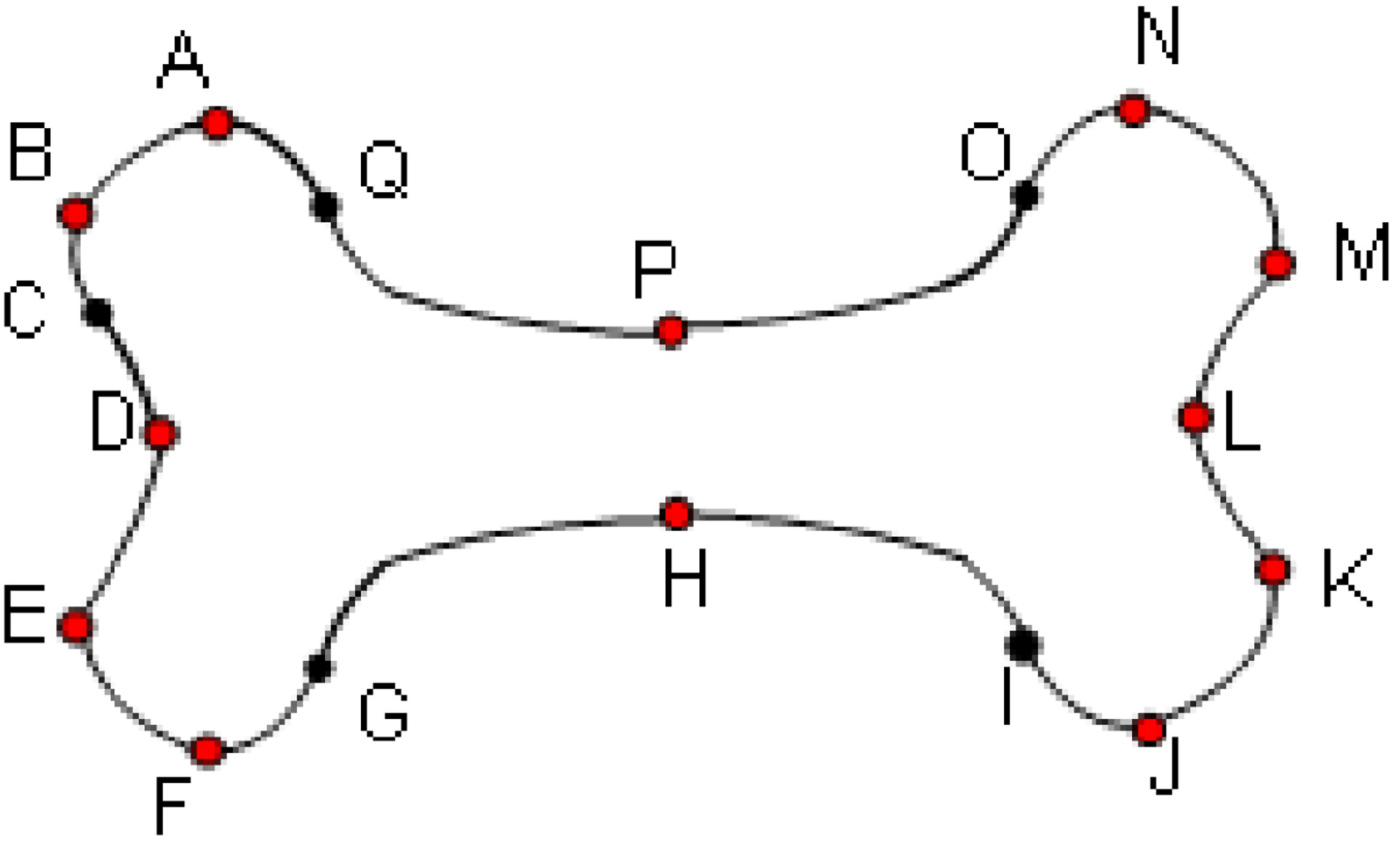
The example of a contour representation. Primitives are separated by dots, both black and white. Sinquads are separated by red dots.

The analyzed contour is divided into primitives. Then, the connected strings of primitives are described in the terms of the shape language characterizations. For instance, the connected fragments, that consist of the primitives belonging to the same quadrant of the Cartesian plane, constitute the sinquad. The transitions between sinquads clearly define the characteristic points for the analyzed contour. Two different neighbouring sinquads constitute a biquad. The key encodes the transitions between successive sinquads, which means that the key describes a sequence of biquads. Thus, the characteristics of the analyzed contour are described by the key. This sequence is analyzed in order to classify types of the recognized contours according to their geometric features. The described approach turned out to be effective in applications to manufacturing [25, 37, 38]. The Jakubowski’s approach, however, turned out not to be the proper tool for bone contour analysis. First of all, bone contours are irregular, in particular, the arcs have variable curvature and, as a consequence, they cannot be segmented into primitives *s*_*ij*_. Therefore, in [13, 14] and [39], substantial generalization has been introduced. Namely, the primitives are defined as the classes of abstraction in an equivalence relation. The relation is defined on the set of all smooth curves that have the same local geometric properties at each point. Let us assume that the analyzed contour is sufficiently smooth, which means that in each of its points, except at most the points in which the primitives join, the tangent line exists and the convexity is determined. This means that at each point the first and the second derivative of the contour that is considered, locally, as the graph of a function, can be calculated apart from the cases in which the denominator zeroes. The properties at the point *u* are described by a four-component vector **c** (*u*) = [*c*_*t*_(*u*), *c*_*c*_(*u*), *c*_*x*_(*u*), *c*_*y*_(*u*)]. The first two components, *c*_*t*_ and *c*_*c*_, encode the information about the first and the second derivative. They can be equal to “+”, “-” and “0” if the value of the derivative is positive, negative or equal to zero, respectively. If the denominator of the derivative zeroes at the point *u*, then the value of the corresponding component of the vector **c**(*u*) is encoded as “V”. The components *c*_*x*_ and *c*_*y*_ encode the information about the increment of *X* and *Y* coordinate along the curve, respectively. In both cases, these components can be positive, negative or equal to zero and they are encoded as “+”, “-” and “0”, respectively. Each maximal fragment of the contour, which has the same aforementioned four characteristics calculated numerically at each point, is treated as a primitive, let us say *p*_*ij*_, *i*, *j* ∈ {1, 2, 3, 4}. Thus, each *p*_*ij*_ can be treated as the equivalent class to which all the segments of lines, which have the same aforementioned characteristics, belong. The index *i* corresponds to geometrical features of the primitives, whereas the index *j* corresponds to the number of a quadrant of the Cartesian plane. It turns out that there exist sixteen equivalence classes (see [14]) and the bi-index of the primitives *p* is defined in such a way that for each *i*, *j* ∈ {1, 2, 3, 4}, *s*_*ij*_ ∈ *p*_*ij*_. Furthermore, the way of contours analysis remains the same as in the original Jakubowski’s method although the primitives are substantially generalized. Let us put an example of encoding of a contour—see Fig 3. The primitives are separated by dots, both black and red, and the sinquads are separated by red dots. Thus, the red dots represent switches. The contour is analyzed counterclockwise. The fragment *AB* is an example of *p*_33_ primitive, whereas the fragment *AD* constitutes the biquad 34 with the switch in the point *B*. The whole contour is represented by the key, which is a string of biquads with an additional logical parameter equal to 1 if the biquad is convex and 0 if it is concave. Thus, starting from the point A, the example contour shown in Fig 3 is encoded as the following key: 341.430.341.411.140.411.121.210.121.231.320.231. It is noticed that, for instance, the fragment *GH* ∈ *p*_41_ has a variant curvature and, as a consequence, it cannot be encoded by using Jakubowski’s primitive *s*_33_.

It should be stressed, however, that even the generalized shape language turns out to be insufficient for classification of the analyzed cases because of the existence of various types of pathological changes. Therefore, the application of hierarchical classification and the application of fuzzy sets algorithm are another improvements of the method. The tree structure of the classification is proposed—see the next section—in the context of analysis of cervical vertebrae contours. In some nodes of the tree the generalized shape language is used, whereas in the others a fuzzy inference algorithm is used.

## Cervical vertebrae contours analysis

**Fig 4:**
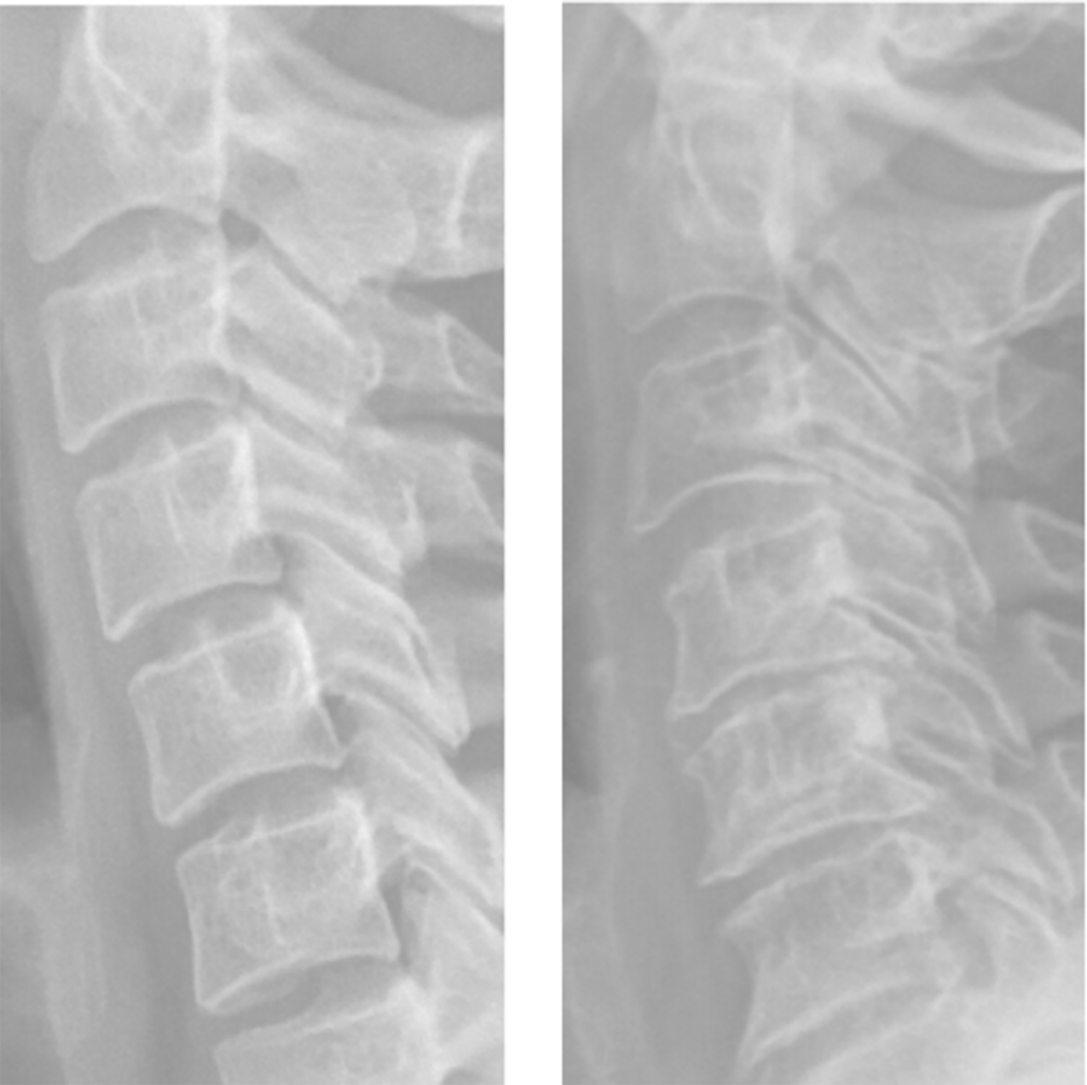
The analyzed vertebrae. On the left the healthy cervical vertebrae, on the right the cervical vertebrae with a new bone formation.

The analyzed vertebra contours have been obtained from X-ray images by using SDA preprocessing method [30] which is dedicated for preprocessing of X-ray medical images. As it has been aforementioned, the approach proposed in this paper is the syntactic one and it differs significantly from the methods used for description contours of vertebrae by other authors—see the state of the art section. The formalism introduced in the previous section was applied to the description and next to recognition of pathological changes of cervical vertebrae. The data set was acquired from the University Hospital in Kraków, Poland. The study protocol was designed according to the guidelines of the Declaration of Helsinki and the Good Clinical Practice Declaration Statement. Special care was taken regarding personal data safety where all images were anonymized before processing. Informed consent for the publication of anonymized clinical images was obtained from the Scientific Committee of the Department of Diagnostic Imaging. As the current study has a retrospective nature, therefore, a consent form for participants was omitted. The data set contained 166 examples of vertebrae, 33 of them were diagnosed as affected by syndesmophyte. In the experiment six vertebrae, denoted by *K*_0_, *K*_1_, *K*_2_, *K*_2_, *K*_3_, *K*_4_, *K*_5_, visible on the X-ray images were analyzed—see Fig 4.

**Fig 5:**
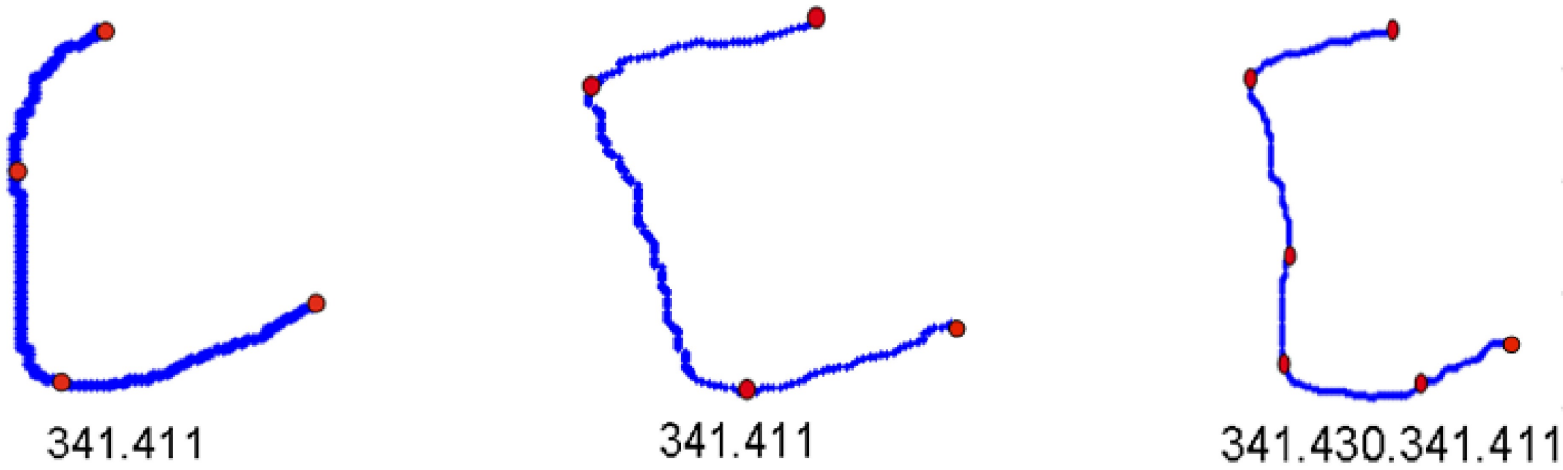
The contours divided into the sinquads. Switches, marked in red, are the points between sinquads that were received as a result of description by primitives *p*_*ij*_. Below each contour, there is a key, which denotes a sequence of transitions between sinquads. After each biquad, there are, additionally, values 0 or 1 which sequence inform about convexity of a given contour.

Since they differ regarding their anatomical structure, each of *K*_*i*_ was treated as a separate set. In the first stage of analysis, the received contours of the vertebrae were described by primitives *p*_*ij*_. This description allowed us to divide a given contour into the sinquads that represented the fragments which belonged to the same quadrant of the Cartesian plane. The transitions between sinquads clearly defined the characteristic points for the analyzed contour. In the case of cervical vertebrae these points, called switches, were essential for its shape—see Fig 5.

To obtain a contour description by using the proposed primitives, the vector values of the components of the vector **c** were calculated. The calculations were carried out on the basis of several neighboring points. In order for small lesions in the outline to be noticed, the number of the points was established in an experimental way. Therefore, the length of the step of numerical calculations of the first and the second derivative was equal to 5 pixels [39]. The accepted step was also equal to the minimal length of primitives. Next, the description of contours by primitives was transformed, according to [13], into the keys. It denoted for the analyzed contour a sequence of biquads which were consecutive transitions between the sinquads. The received keys created equivalent classes that in some cases were sufficient for the process of recognition. It means that they coincided with the expected classification of an initial contour set. If the received equivalent classes do not distinguish the analyzed cases correctly, the fuzzy analysis, based on additional features of biquads, is introduced. In the case of cervical vertebrae, the received keys are presented in Table 1.

**Table 1:** The received keys and equivalent classes in the analyzed data.

Keys	Equivalent classes
341.411	healthy vertebrae or with early pathological changes
341.430.341.411	
341.430.341.430.341.421.210	vertebrae with serious pathological changes
341.430.311.140.411	
341.430.341.430.341.411.121.210	

**Fig 6:**
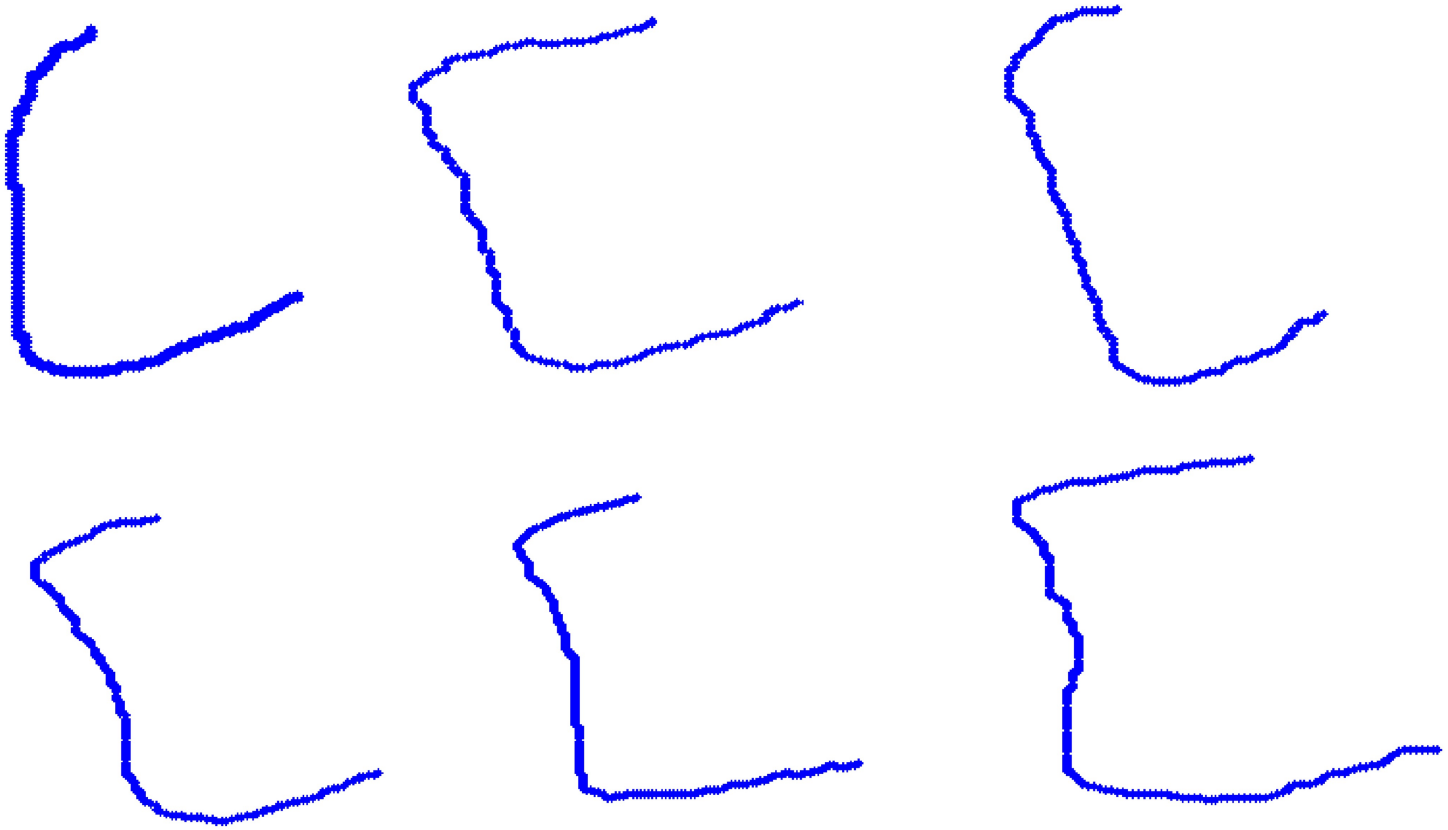
The contours of healthy vertebrae. The contours have been received by using SDA algorithm. In the first row, there are *K*_0_, *K*_1_ and *K*_2_ described by string 341.411. In the second row, there are *K*_3_, *K*_4_ with the description 341.411 and *K*_5_ described by string 341.430.341.411.

**Fig 7:**
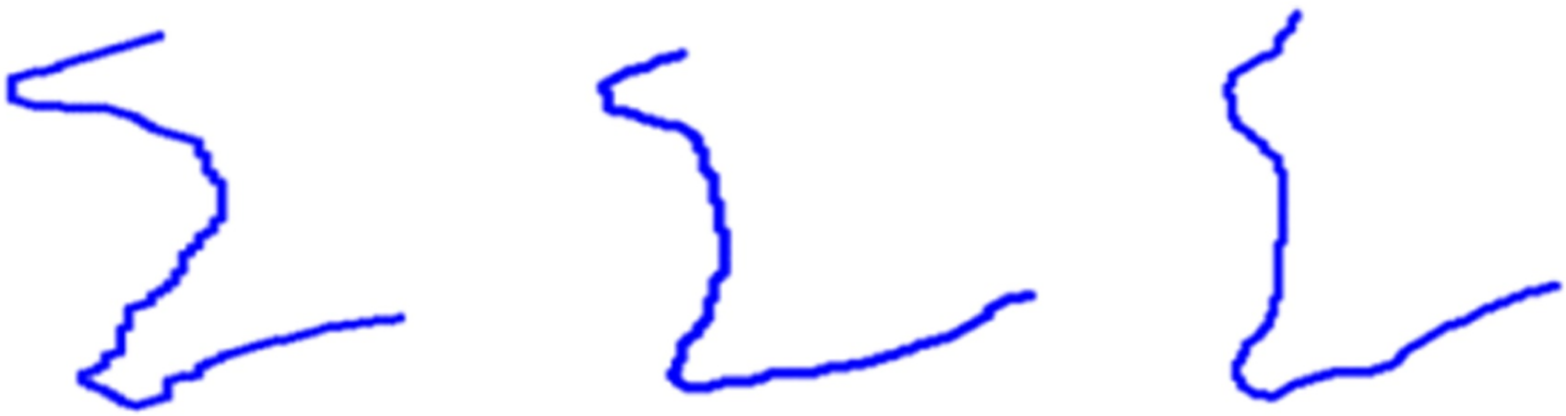
The contours of the vertebrae with early pathological changes. The contours of the vertebrae *K*_2_, *K*_3_ and *K*_4_ from Fig 4 with pathological changes received by SDA algorithm. The first and third ones are described by string 341.430.341.411. The second one is described by string 341.411.

**Fig 8:**
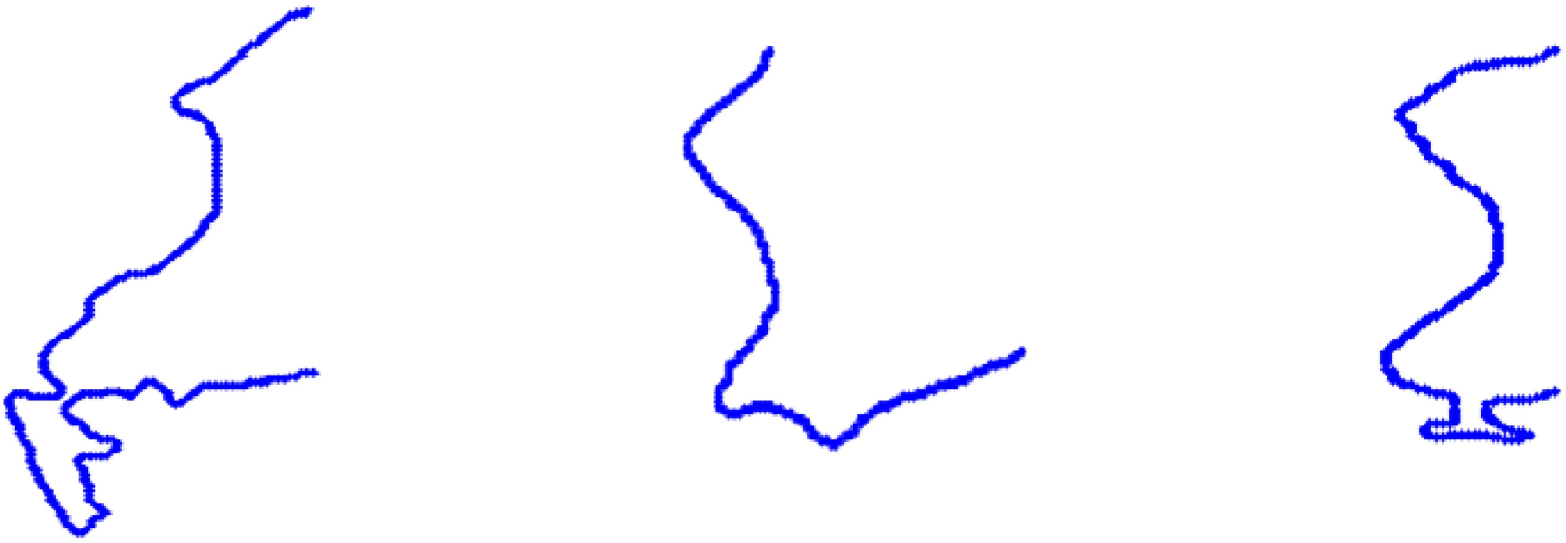
The contours of the vertebrae with serious pathological changes. Each vertebra is described by untypical keys—see Table 1.

The description of vertebrae by strings of biquads allowed us to distinguish the healthy ones—see Fig 6—and the ones with early changes—see Fig 7—from the strongly affected vertebrae—see Fig 8.

**Fig 9:**
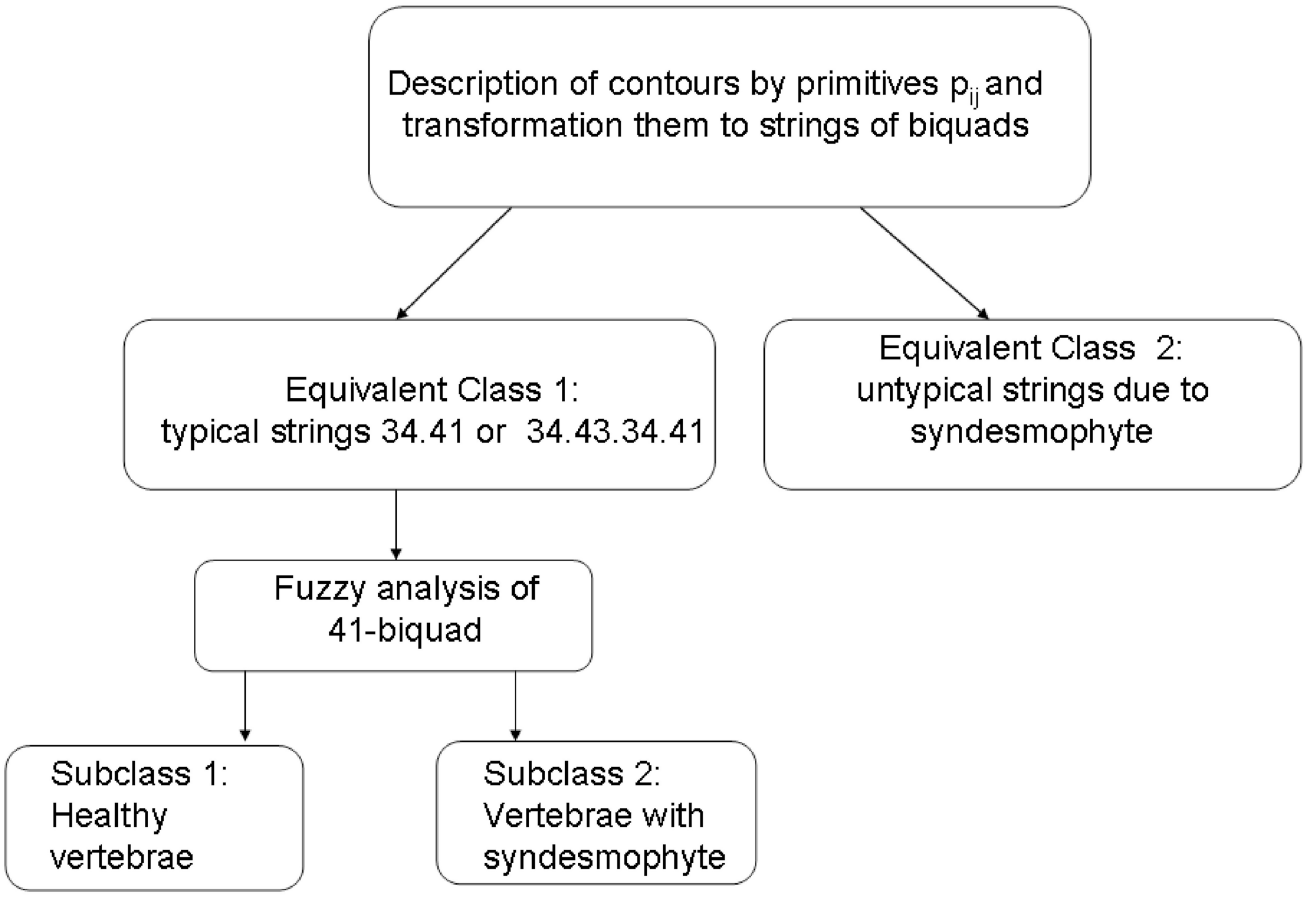
The diagram of the proposed hierarchical method of vertebrae contours analysis.

By early changes, it is understood that a given contour is still described by a typical string of biquads but it can be distinguished from healthy ones due to the features of biquads. Thus, the equivalent class of vertebrae with serious pathological changes does not contain subclasses. Nevertheless, the equivalent class of the healthy vertebrae and the one with early pathological changes consists of two subclasses—see Table 1. Therefore, the fuzzy analysis has to be applied only to this case. The diagram of the proposed method is presented in Fig 9.

**Fig 10:**
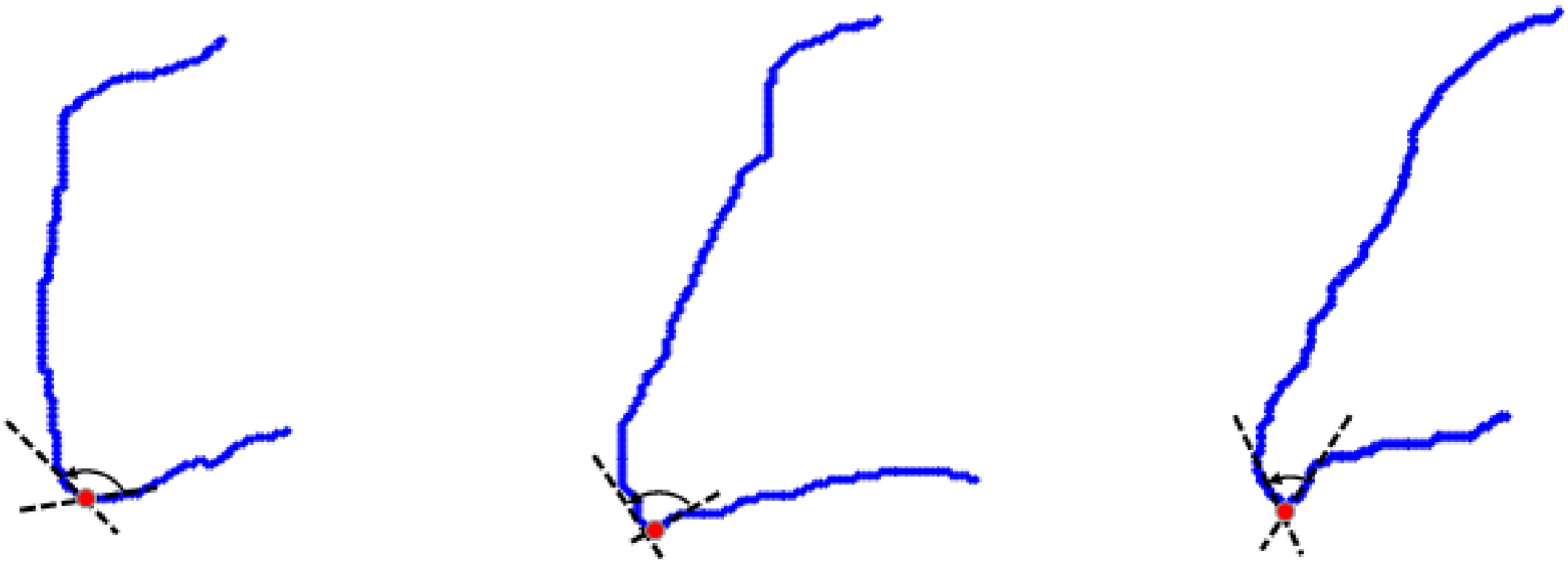
The determining of the angle *α*. The examples of vertebrae with an angle *α* between 4−*sinquad* and 1−*sinquad* are marked. Auxiliary straight lines help to indicate the angle. From the left: a healthy bone, a bone with a small lesion, a bone with the advanced lesion.

The values 0 and 1 that appear after each of the biquad inform about convexity of the given contour. The lesions that should be found are indicated by the switch between biquads 34 or 41. Angles *β* and *α* between 3−*sinquad* and 4−*sinquad* as well as 4−*sinquad* and 1−*sinquad* are the features that allow us to distinguish between healthy vertebrae and vertebrae with syndesmophyte. Both classes, healthy vertebrae and vertebrae with syndesmophyte, are treated as fuzzy sets. The values of *α* and *β* are determined on the basis of the values of components *p*_*ij*_, calculated in the previous stage. In this paper the lesion placed in the lower part of a vertebra was considered, thus the angle between 4−*sinquad* and 1−*sinquad* was taken into account. The angle *α* was determined by the last minimal primitive in the 4−*sinquad* and the first minimal primitive in the 1−*sinquad*. Three examples of vertebrae: a healthy one, with a small lesion and with a distinct lesion are shown in Fig 10.

**Fig 11:**
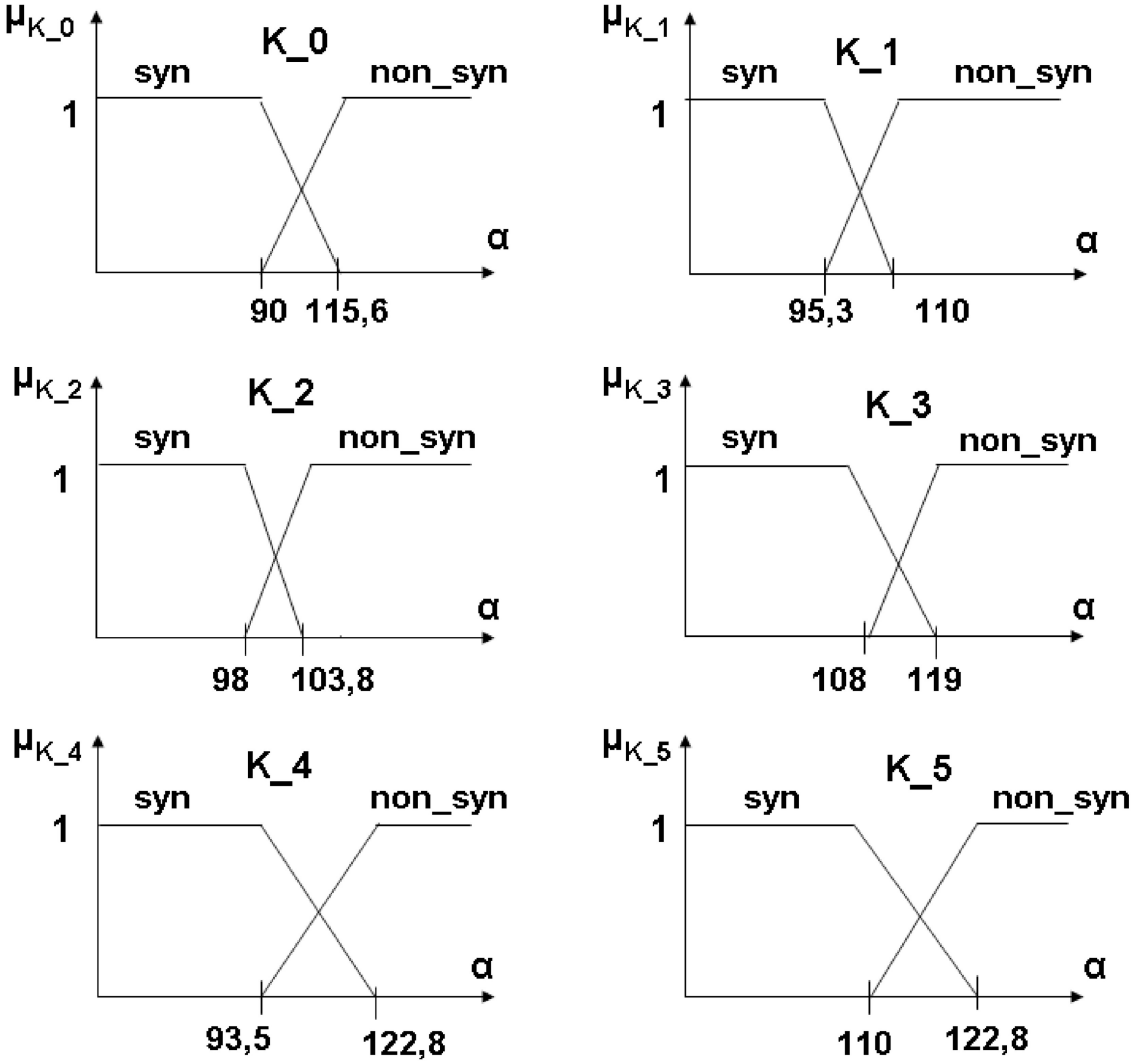
The graphs of the membership functions. The membership functions are defined for each specified class i.e. *K*_0_, *K*_1_, *K*_2_; *K*_3_, *K*_4_, *K*_5_. The angle *α* is the angle between 4−*sinquad* and 1−*sinquad*.

For each set *K*_*i*_ two membership functions were constructed. The functions determined whether a considered vertebra is pathological or not. The trapezoidal functions as membership functions were used with the parameters established in a statistical way—see Fig 11.

Modifiers for the received membership functions were calculated in the way described in [40]. According to it, the area under each graph of a membership function was divided as follows:

$$\;S_{mfalse}=\frac{(b-a)n}{4},\;\;\text{for}\;n\in \left[0,1\right],$$

$$\;S_{mtrue}=\frac{\left(b-a)(n-1\right)}{n},\;\;\text{for}\;n\in \left[2,+\infty \right)$$

and

$$\;S_{t}=\frac{(b-a)n}{4},\;\;\text{for}\;n\in \left[1,2\right],$$

The value *n* corresponds to the modifier *m*. The interval [2, +∞) characterizes the terms with a linguistic *truth* value greater than or equal to *true*, whereas [0, 1] characterizes the terms with a linguistic *truth* value less than or equal to *false*. The interval (1, 2) allows all the possible variants between *true* and *false* to be expressed. Thus, for the membership function $\mu_{syn}^{0}$, which identified vertebrae in the class *K*_0_ with syndesmophyte, the following areas were calculated: the one that defines the label *false*:

$$\;S_{mfalse}=\frac{(115.6-0)\cdot 1}{4}=28.9,\;\;\text{for}\;n=1,$$

and the one that defines the label *true*:

$$\;S_{mtrue}=\frac{\left(115.6-0)\cdot (2-1\right)}{2}=57.8,\;\;\text{for}\;n=2.$$

From calculation, it results that a given vertebra belongs rather to the class of vertebrae with syndesmophyte if the value of its membership function $\mu_{syn}^{0}\left(\alpha \right)\geq 0.58,$ whereas $\mu_{syn}^{0}\left(\alpha \right)\leq 0.29$ means that it does not belong to this class. Additionally, a dilation operator supplies the following rules:

if $\mu_{syn}^{0}\geq 0.76$ then a vertebra clearly belongs to the class with syndesmophyte;
if $0.76=\sqrt{0.58}>\mu_{syn}^{0}\geq 0.58$ then a vertebra almost belongs to the class with syndesmophyte;
if $0.58>\mu_{syn}^{0}>0.29$ then the classification is unclear;
if $0.29\geq \mu_{syn}^{0}$ then a vertebra does not belong to the class with syndesmophyte.

In turn, for the membership function $\mu_{non\_syn}^{0}\left(\alpha \right),$ which identified the healthy vertebrae, the calculated areas under this function is:

$$\;S_{mfalse}=\frac{(180-90)\cdot 1}{4}=22.5,\;\;\text{for}\;n=1,$$

the ones that defines the label *true*:

$$\;S_{mtrue}=\frac{\left(180-90)\cdot (2-1\right)}{2}=45,\;\;\text{for}\;n=2.$$

The received rules have the following form:

if $\mu_{non\_syn}^{0}\geq 0.67$ then a vertebra clearly belongs to the class without pathological changes;
if $0.67=\sqrt{0.45}>\mu_{non\_syn}^{0}\geq 0.45$ then a vertebra almost belongs to the class without pathological changes;
if $0.45>\mu_{non\_syn}^{0}>0.23$ then the classification is unclear;
if $0.23\geq \mu_{non\_syn}^{0}$ then a vertebra does not belong to the class without pathological changes.

Analogically, the fuzzy rules for the other classes were created. The received results of classification of some vertebrae are presented in Table 2.

**Table 2:** The results of the classification.

No.	$\mu_{s}^{0}$	$\mu_{ns}^{0}$	$\mu_{s}^{1}$	$\mu_{ns}^{1}$	$\mu_{s}^{2}$	$\mu_{ns}^{2}$	$\mu_{s}^{3}$	$\mu_{ns}^{3}$	$\mu_{s}^{4}$	$\mu_{ns}^{4}$	$\mu_{s}^{5}$	$\mu_{ns}^{5}$
1	0	1	0	1	0	1	0	1	0.13	0.87	x	x
2	0	1	0	1	0	1	0	1	0	1	x	x
3	0.14	0.86	0	1	0	1	0	1	0	1	x	x
4	0	1	0	1	0	1	0	1	0	1	x	x
5	0	1	0	1	0	1	0	1	0	1	0	1
6	0	1	0	1	0	1	0	1	0	1	0	1
7	0	1	0	1	0	1	0	1	0	1	x	x
8	0	1	0	1	0	1	0	1	0.02	0.98	0.2	0.8
9	0.03	0.97	0	1	0	1	0	1	0	1	0	1
10	0.38	0.62	0	1	0	1	0	1	0	1	0.38	0.62
11	0	1	0	1	0	1	0	1	0	1	0	1
12	0	1	0	1	0.48	0.52	0	1	0	1	x	x
13	0	1	0	1	0	1	0	1	0	1	0	1
14	0	1	0	1	0	1	1	0	1	0	0	1
15	0	1	0.86	0.14	1	0	1	0	1	0	x	x
16	0	1	0	1	0	1	1	0	0.78	0.22	0.99	0.01
17	0	1	0	1	0	1	1	0	1	0	0	1
18	x	x	0	1	0	1	0.62	0.38	0.9	0.1	0.34	0.66
19	0	1	0	1	0	1	1	0	0	1	0.99	0.01
20	0	1	0.35	0.65	0.84	0.16	1	0	0	1	x	x
21	0.8	0.2	0	1	0	1	1	0	1	0	0	1
22	x	x	1	0	1	0	1	0	1	0	x	x
23	1	0	0	1	0	1	1	0	1	0	x	x
24	0	1	1	0	0	1	0	1	0	1	0	1

The symbol x means that a vertebra is not visible at the X-ray image.

**Fig 12:**
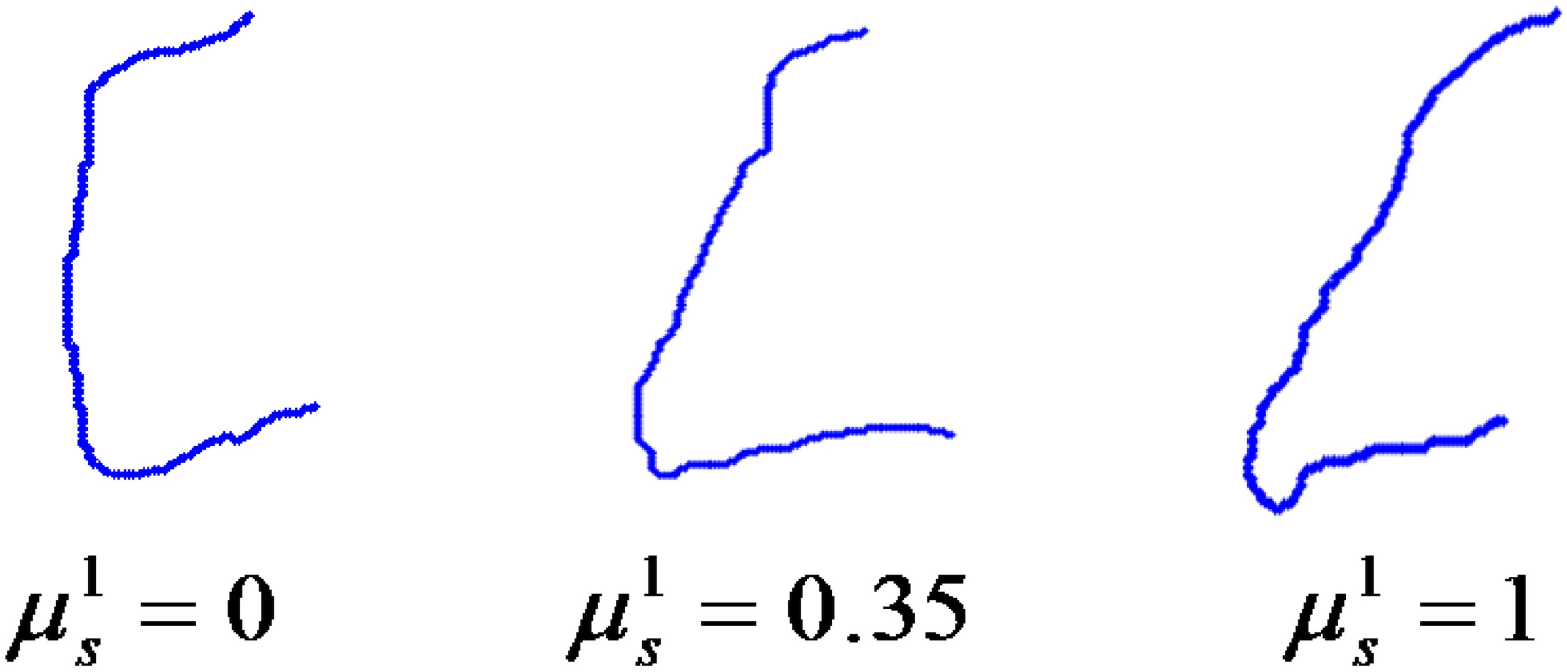
Three examples of vertebrae from *K*_1_ and the values of the membership functions to the class with syndesmophytes. The values are calculated for vertebrae from the set *K*_1_ which are presented in Fig 10.

If the value of a membership function is equal to 1 or around 1, then a given vertebra fully belongs to the specified class. If the value is around 0, then it does not belong to this class. In all the cases, the received results coincide with the classification made by an expert but, additionally, the information about diversity in both considered classes was received. In the case of the healthy class, this diversity was small and it resulted from anatomical differences. In the case of the class with syndesmophytes, the diversity was larger and it resulted from the size of pathological changes. In Fig 12 there are three examples of vertebrae from the set *K*_1_ with different values of membership functions $\mu_{syn}^{1}$. The aim of this paper was to show that the proposed method allows us to classify all vertebrae correctly. If we adopt the simplest rule, namely that a given vertebra belongs to the class for which it has the greatest value of the membership function, then we get 100% correctness. Of course, the different degree of belonging to the class with syndesmophytes is very important in terms of the possibility of assessing the progress of a disease.

## Concluding remarks

The method of hierarchical analysis of contours of vertebrae, presented in this paper, is based on syntactic and fuzzy pattern analysis. It should be mentioned that the method is analogous to the method that was applied by the authors to the analysis of contours of finger bones—see [11, 12, 15] and, first of all, [13]. Though it is related to a few streams of studies which concern the analysis of bone contours, including the contours of vertebrae, the approach, proposed in this paper, is based on a different basis and it is a novel one. The method achieved 100% accuracy provided that the pattern was classified to this class which had a greater value of a membership function. This means that every pattern has been classified as a healthy bone or a bone with pathological changes in the same way, by the algorithm and by an expert. As it has been aforementioned, in the introduction, the proposed method is considered not only in the context of detection of pathological changes in bones but also in the context of the possibility of assessing the disease progress. The fact that the value of a membership function depends on the size of a pathological change is a good starting point for creating a tool which allows us to infer about the progress of the disease. This is planned to be the topic of our future work.

It should also be mentioned that the proposed method is not limited to medical application. It can be potentially effective in each problem in which contour analysis according to its shape properties is one of the key tasks. Scene analysis by cognitive vision module of autonomous robots can be put as an example of such a problem [41–43]. In the mentioned papers the industrial scene is represented by using only polygonal shapes. It should be stressed, however, that such representation can be insufficient in, for instance, oriental cities, where a lot of domes exists. In such cases both curvilinear and line segments are necessary to represent the objects and, for this reason, the proposed approach can be applied in all its length. The contour representation and analysis in the context of the scene analysis is also examined for solving superimposition problems for aerial photographs in an onboard computer vision systems [44, 45]. In this problem contours of natural objects such as river banks and contours of roads are analyzed in order to the work out correction methods for navigation parameters. The landscape representation and analysis in the context of the autonomous robot navigation is also studied in the context of representing the contours of obstacles [46]. The proposed method is potentially applicable to this task as well.
